# Can a prolonged healing pressure injury be benefited by using an AI mattress? A case study

**DOI:** 10.1186/s12877-024-04900-x

**Published:** 2024-04-02

**Authors:** Tung Fang Ni, Jyh-Liang Wang, Chih-Kuang Chen, De Fen Shih, Jeng Wang

**Affiliations:** 1 Chang Gung Memorial Hospital, Nursing home, Taoyuan, Taiwan; 2 Department of Electronic Engineering, Ming Chi University of Technology, New Taipei city, Taiwan; 3 Department of Physical Medicine and Rehabilitation, Chang Gung Memorial Hospital at Taoyuan, Taoyuan, Taiwan; 4 School of Medicine, Chang Gung University, Taoyuan, Taiwan; 5 eBio Technology Inc, New Taipei city, Taiwan; 6 Geriatric & Long-term Care Research Center, Chang Gung University of Science and Technology, 261 Wen-Hwa 1 Rd, Kwei-Shan, Tao-Yuau, Taoyuan, 333, Taiwan; 7 Chang Gung Memorial Hospital, Keelung, Taiwan

**Keywords:** Pressure injury, Nursing home residents, Artificial intelligence mattress

## Abstract

**Background:**

Pressure injuries are a common and serious issue for bedridden residents in long-term-care facilities. Areas of bony prominences, such as the scapula, sacrum, and heels, are more likely to develop pressure injuries. The management of pressure injury wounds include dressing changes, repositioning, away from moisture, decreasing the occurrence of friction and shear, and more. Some supportive surfaces are also used for pressure injury cases such as gel pads, alternating pressure air mattresses, and air-fluidized beds. The aim of this case study was to determine whether the use of an artificial intelligent mattress can improve a nursing home resident with prolonged pressure injury.

**Case presentation:**

A retrospective study design was conducted for this case study. A 79-year-old male developed a pressure injury in the sacrum. His pressure injury was initially at stage 4, with a score of 12 by the Braden scale. The PUSH score was 16. During 5.5 months of routine care plus the use of the traditional alternative air mattress, in the nursing home, the wound stayed in stage 3 but the PUSH score increased up to 11. An artificial intelligence mattress utilizing *3D InterSoft* was used to detect the bony prominences and redistribute the external pressure of the skin. It implements a color guided schematic of 26 colors to indicate the amount of pressure of the skin.

**Results:**

The wound size was decreased and all eczema on the resident’s back diminished. The PUSH score was down to 6, as the artificial intelligent mattress was added into the routine care. The staff also reported that the resident’s quality of sleep improved and moaning decreased. The hemiplegic side is at greater risk of developing pressure injury.

**Conclusions:**

This novice device appeared to accelerate wound healing in this case. In the future, more cases should be tested, and different care models or mattress can be explored.

**Supplementary Information:**

The online version contains supplementary material available at 10.1186/s12877-024-04900-x.

## Background

Pressure injury (PI) is a type of localized skin damage caused by moisture, friction, shear, or prolonged pressure, especially in areas of bony prominences, such as the sacrum, coccyx, and heels [1, 2]. There are many different ways to classify PI stages. The most common classification for PI are the four stages and the unstageable classification [3]. PI stages are defined on the basis of the severity of skin, tissue, and muscle damage. Particularly, once a PI has reached Stage 3, extending through the skin into deeper tissue and fat but do not reach muscle, tendon, or bone, the treatment process becomes protracted [4, 5] Interventions for PI prevention and treatments include support surface, regular repositioning, appropriateness of nutritional status, skin moisturization, and others [2, 6]. Gel pads, alternating pressure air mattresses (APAMs), and air-fluidized beds (AFBs) are support surfaces frequently used for PI cases [7, 8]. The purpose of using support surfaces is to maximize the body surface area where the body contacts a bed, distribute the body weight, lower the pressure, reduce shearing forces, and control the local microclimate [7, 9]. However, the selection of appropriate mattresses in clinical practice remains difficult since most support surfaces recommended are either by expert opinion or via low levels of evidence [7, 10, 11]. Normal capillary closure pressure ranges from 20 mmHg to 40mmHg, with 32 mmHg considered the average [4, 5]. If constant pressure on capillary arteries is prolonged, and external pressure exceeds the arterial capillary pressure of 32 mm Hg the blood flow can be impeded, so wound healing may be drastically slowed [5]. Especially for those with PI at Stage 3 or Stage 4, high pressure can prevent blood flow from entering the wound site [5]. Therefore, in clinical settings, an APAM is often used in many facilities because of its low cost and easy access. An APAM contains a pump and a celled bed with low air pressure inside. Usually, every 2 or 3 cells is a set, and one air cell releases its pressure, the other one (two) remains the pressure, and the redistribution is based on a time schedule, not on skin pressure [7, 11, 12]. In contrast, AFBs are heavy and expensive. AFB uses warm air under pressure to set tiny silica or glass beads, called microspheres, in motion, which emulates the movement of fluid. When the resident is placed in the bed, his body weight is evenly distributed over a large surface area, which creates a sensation of “floating “. Therefore, they tend to be used in burn units in acute hospitals. In this study, a new kind of mattress was used with an artificial intelligence (AI) application, *3D InterSoft* [13], which makes it possible to calculate the pressure on bony prominences in nursing residents or residents and redistribute the pressure automatically. Three major functions are integrated to the AI mattress, which includes the ePad fabric flexible sensor, the air bubble array, and the intelligent control box. The ePad fabric flexible sensor array and the air bubble array are separated into 1080 (20 × 54) cells in the mattress respectively, and they are connected to each other. The dimension of a single-pressure sensor is 2.2 × 2.2 cm2 with a spacing of 3 cm to the neighboring sensor. When the resident lies on the bed the first time, the mattress does a preliminary scan to identify the baseline. The AI in the mattress locates the areas of bony prominences and actively compares the aforementioned data with its’ database. Furthermore, the Intelligent control box drives the air bubble array to adjust the softness of the mattress. The mattress will then alter and relieve the pressure areas. As a result, pressure injuries can be prevented.

**Fig. 1:**
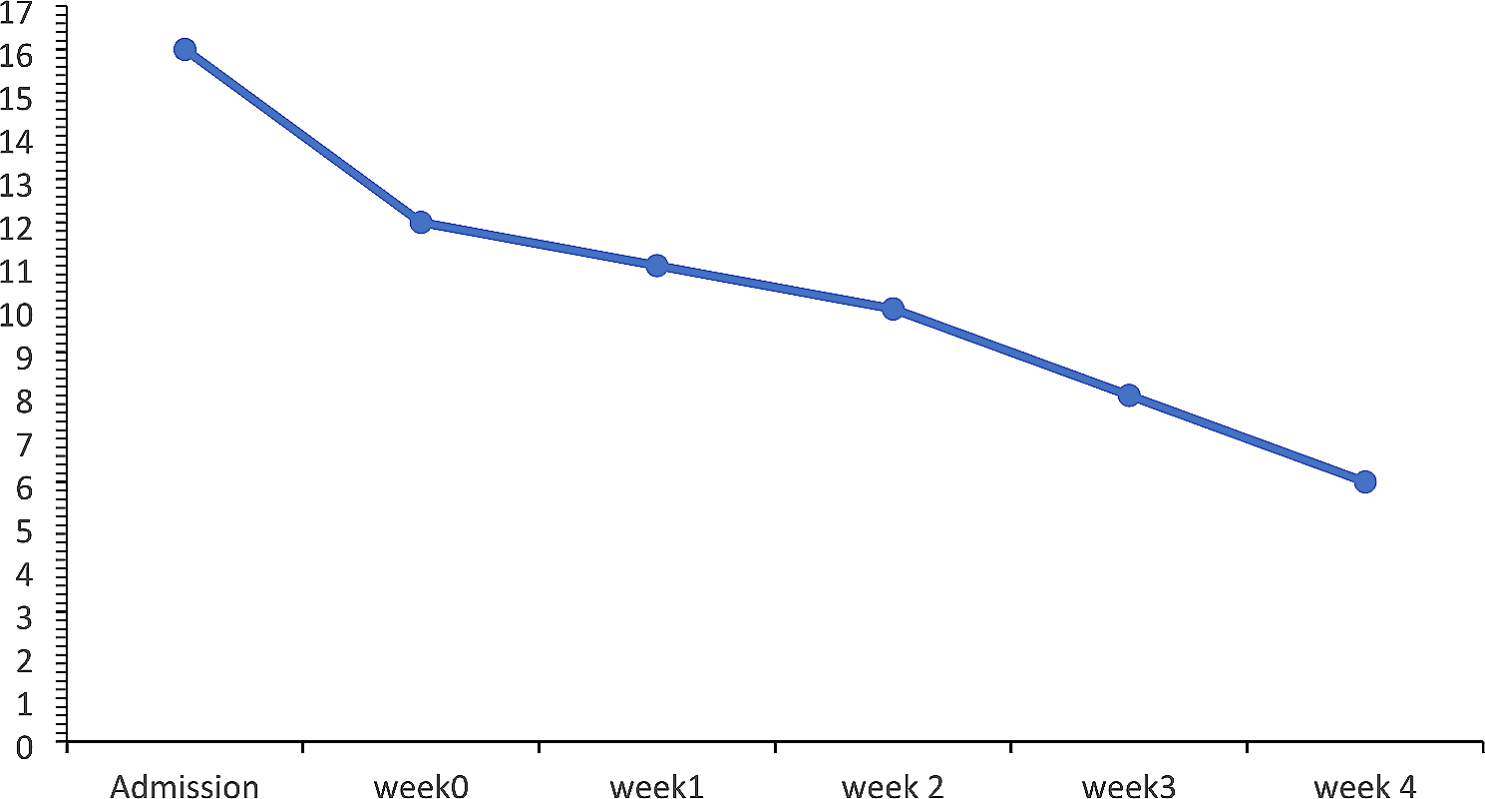
Changes in PUSH scores overtime PUSH score

The mattress completes a reading with modifications to the cells every 60 min or every time the resident switches position. Each change in the cell provides more than 15% of decreased pressure pertaining to the original area. Moreover, the AI uses a color guided schematic of 26 colors, (Fig. 1) each with a representative gradient color to indicate the levels of pressure. According to the data obtained from the pressure sensor after the user lies down, 5% of the highest values are designated as the red portion, and 10% of the lowest values are designated as the white portion. Thus, with a redistribution of pressure the schematic changes accordingly. Nursing staff can monitor the pressure by observing the color scheme on the screen of the intelligent control box. Therefore, the aim of this case study was to determine whether the use of an AI mattress can improve a nursing home resident with PI.

## Method

This is a retrospectively designed case study. The case was the resident of an independent nursing home in Taiwan. All the data such as PI wound size, skin condition, and staff record were collected anonymously based on the chart review from Nov. 1., 2022 to Nov30, 2022. The pressure on the area of bony prominence was recorded by the AI mattress automatically very second. All the data were coded and analyzed by using the software of SPSS version 25.0. The descriptive statistical analysis and paired-t test was used based on the aim of this study.

**Table 1:** Pressure of bony prominence (risky) site

	Left Scapula	Right Scapula	Sacrum	Left Hip	Right Hip
Mean ± SD	42.76 ± 5.70	38.55 ± 8.09	35.70 ± 9.78	56.76 ± 15.08	47.66 ± 10.64
median	42.21	98.85	35.02	50.91	44.03
Max	54.11	72.45	53.46	88.38	80.42
Min	26.52	22.90	18.24	38.06	30.48
Range	28.52	49.55	35.21	50.33	49.94
Lower 95%CI	40.55	35.41	31.92	50.92	43.53
Higher 95%CI	44.97	41.68	39.50	62.61	51.78
	Left scapula-Right scapula			Left hip-Right hip	
t-value	2.35*			9.45***	

Note: *p <.05, **p <.01, ***p <.001

## Ethical approval

This case study was approved by the Chang Gung Foundation Institution Review Board (approval No: 202201641B0). The Helsinki Declaration was followed throughout the study. The researcher explained the study’s purpose, risk, and benefits prior inclusion. Written consent for data collection and publication was obtained by the surrogate of the subject. The surrogate of the participant has signed the informed consent for publication of identifying information/images. This case study was reported according to CARE case report guidelines.

## Case presentation

The case recruited in this study was a 79-year-old male with a history of multiple diseases, including heart disease and myocardial infarction, who experienced long-term bedridden status after a heavy stroke. He was admitted to a nursing home because of left-sided hemiplegia and the need for positional changes. Although he was conscious, he could not express himself verbally, so he sometimes made grunting sounds to notify staff for diaper changes. In nutrition, 2100 kilocalories were given daily. Six bottles of Ensure® and 600 cc-800 cc of high protein drinks were administered through a nasogastric tube with four equal doses per day. His body mass index was 24 kg/m^2^.

On admission, A PI developed on his sacrum with a size of 6.5 × 3.5 × 3 cm^3^, and his PI was classified as Stage 4 according to the NPUAP The Braden scale was used to evaluate the risk of PI concurrently. In the Braden Scale his sensory perception, nutrition, and moisture were all scored 3, but friction and shear, activity, and mobility were scored 1 by the nursing staff. As a result, the total score was 12, indicating a high risk of PIs [5]. Moreover, the Pressure Ulcer scale for Healing (PUSH) was used to evaluate the healing status of PI [14]. The sub-score for the length x width was 9, for exudate amount was 3, and for tissue type was 4. The total score of PUSH was 16(Fig. 1). In addition, the staff reported several eczemas on the resident’s back and the resident moaned most of the time. Three major strategies were identified as the routine for PI care in this nursing home: first, changing position every 2 h allows for a decrease in pressure caused by being bedridden; second, the diaper and clothes were checked every two hours and changed if needed to ensure a relatively dry condition for the skin. Finally, a wet dressing wound change with antibiotic ointment is done once a day.

**Fig. 2:**
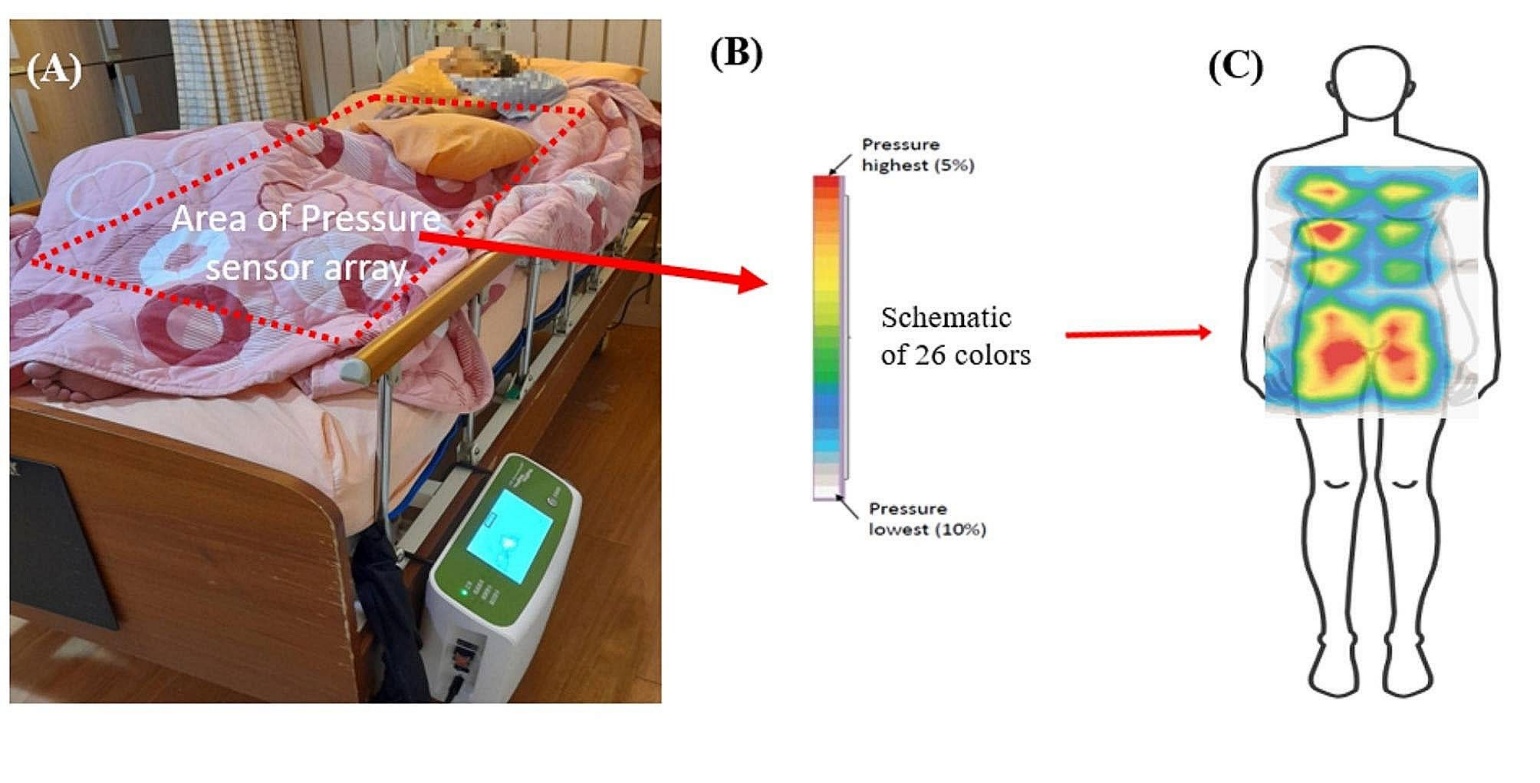
Picture of real situation for the case in the Nursing Home (**A**) an AI mattress on bed with a box aside the bed (**B**) a color guided schematic of 26 colors to indicate the levels of pressure (**C**) an colored image of the pressure showing on the screen of the box. As the AI mattress scans the case, all bony prominences are identified

Improvement of his PI was one of the major concerns for admitting him to this nursing home, so a traditional APAM was used to relieve the pressure of being bedridden. Five months later, the wound size had decreased to 2.5 × 2 × 3 cm^3^.The PUSH score was down to 11 but no further improvement was observed. Consequently, an air mattress with the AI application was used to replace the traditional APAM; wound dressing and repositioning remained as before. The size of the AI mattress was similar to that of the traditional APAM but had an active pressure sensor array that ensured the accuracy of positioning. The pressure redistricting process is a dynamic process; this AI mattress can be programmed to automatically detect and calculate the pressure every second. The AI device uses the result from the first-time scan as the baseline or compare the aforementioned data to locates the areas of bony prominences and showed in a 26-colored schematic (Fig. 2). The red portion indicated the area with highest risk to develop PI. As a result, the pressure would be redistributed by the AI mattress.

**Fig. 3:**
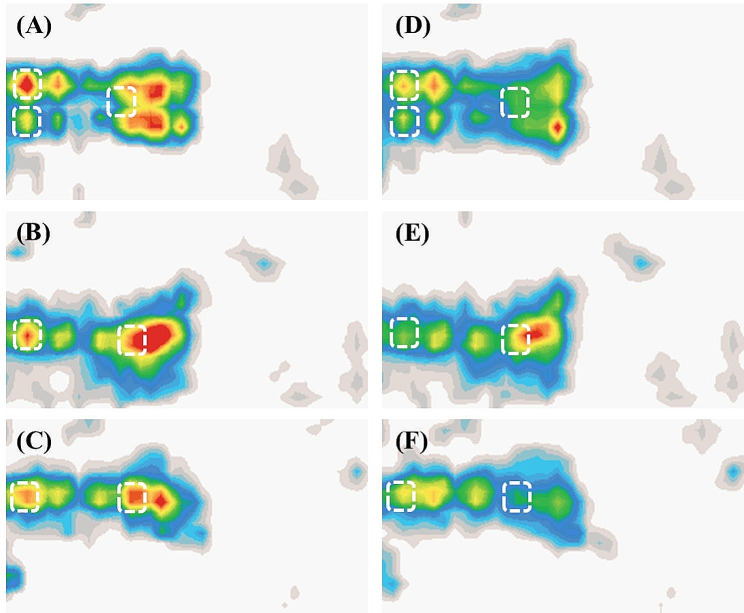
Using the AI device to detect bony prominences and to redistribute the pressure Panel (**A**), (**B**), (**C**) are pressure of lower extremities in different positions, flat, left, and right postures, respectively. The white dotted lines (square)were bony prominences Panel(**D**), (**E**) and (**F**) are pressure of lower extremities in different positions, flat, left, and right postures, respectively, after the pressure was redistributed The pressure in white squares decreased once the pressure was redistributed by the AI device

Therefore, Fig. 3 (A) to (C) are demonstrating how different colors were used to highlight the pressure level in different positions, with red indicating high pressure and thus a higher risk of PI development, while white indicated a lower pressure. Figure 3 (D) to (F) are displaying the redistributed pressure once the risk area was identified. Figure 3(A) shows the body supine, Fig. 3(B) shows the resident lying on his left side, and 3(C) shows the resident lying on his right side. A sensory array was used to scan and record the pressure data and connected to each cell. AI was set to automatically calculate skin pressure and presented in colored schematic. Figure 3(C), (D), and (E) show that the AI mattress automatically redistributed pressure as AI compared with the aforementioned data and identified risky areas.

**Figs. 4:**
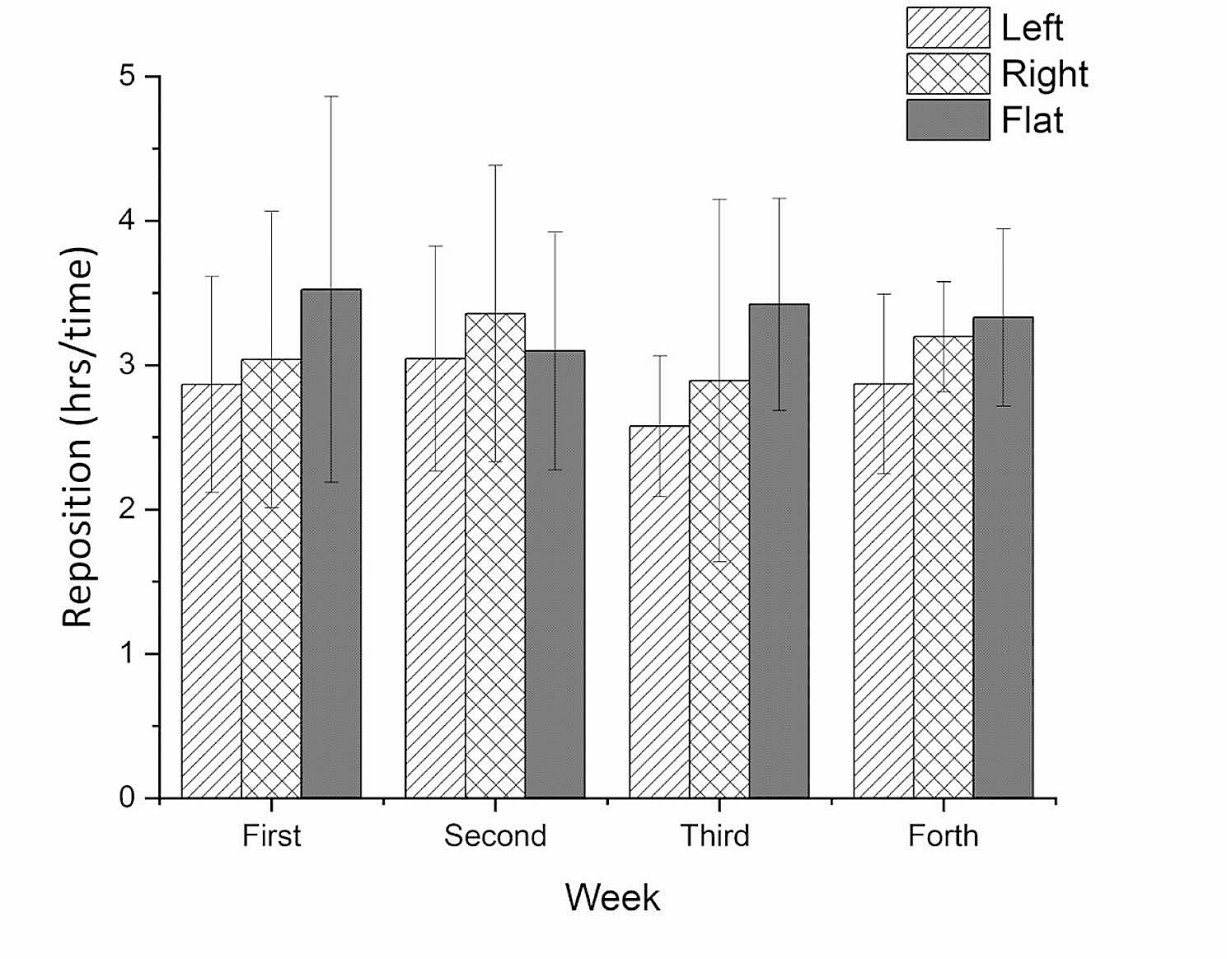
Average hours of lying on bed per time in different positions by the AI mattress overtime

**Figs. 5:**
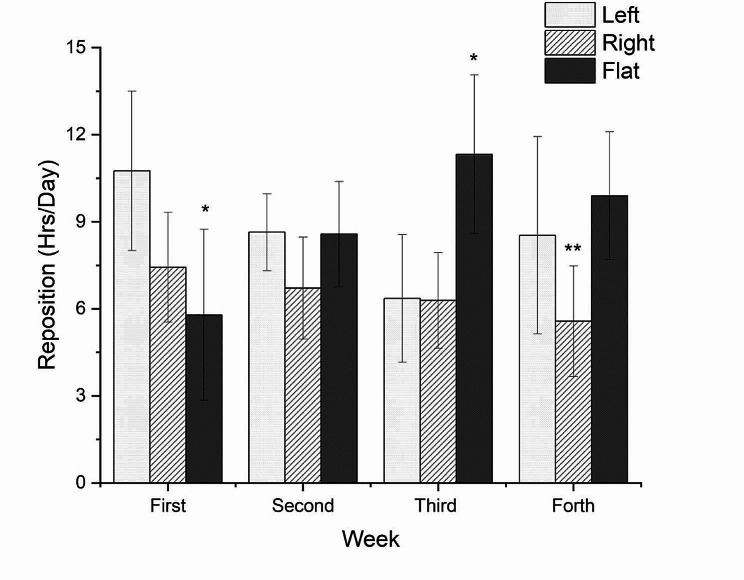
A total hour of lying on bed per day in different positions by the AI mattress overtime

The case used the AI mattress for 4 weeks. Figure 4 shows that repositioning by the nursing home staff occurred every 3.10 ± 0.25 h. In total, the time lying on the bed increased from 5.79 ± 2.95 h per day in the first week to 9.89 ± 2.21 h in the 4th week (Fig. 5). Regarding the total time spent on the bed in different positions, the time spent supine or sitting up at 30° was the highest (8.89 ± 2.36 h/day), followed by the left side (8.57 ± 1.79 h/day) and right side (6.50 ± 0.78 h/day). Due to the use of the AI device, no new PIs developed, and the size of the original PI decreased. Interestingly, left-sided pressure in the scapula and hip was significantly higher than right-sided pressure (*p* <.05) during both day and night (Table 1). Photographs of the PI wounds were taken and measured weekly. The size of the wound was 2 × 2.5 × 3.2 cm^3^ at the start of using the AI mattress. Four weeks later, the size had reduced to 2 × 1 × 1.2 cm^3^ (stage 2), and the score of the PUSH was down to 6(Fig. 1).

According to the NPUAP, repositioning every 164–240 min, is recommended to prevent PIs [2, 15, 16]. However, the reposition routine in this nursing home and most health institutions in Taiwan still remains with a frequency of every 2 h. In Fig. 4, the AI data showed that the average time between repositioning was 3.11 h, much longer than the required 2 h in this nursing home. Over these 4 weeks in which the AI mattress was used, the average time for lying supine or sitting on the bed at 30° (with a stuffed pillow behind the resident’s back) was always longer than 3 h; the average time for lying on the right side was approximately 3 h, but lying on the left side was always less than 3 h.

## Discussion

PIs are mainly caused by long-term pressure on weak skin, especially in areas of bony prominence [17]. According to the Braden scale, sensory perception, moisture, activity, mobility, friction, and shear are the 6 major parameters used to measure the risk of PI [6, 17, 18]. The case presented in this study is still at high risk of developing PI, since in addition to multiple illnesses and issues of immobility due to hemiplegia, the resident suffered from urinary incontinence. Consequently, poor recovery from a PI wound was foreseeable [6, 19]. Additionally, in this case, use of passive changing of positions every 2 h and wet dressing wound care with antibiotic ointment may not fully follow the international guideline for prevention and treatment of pressure ulcer of PI [6, 18]. Treatments such as negative pressure oxygen therapy or wound debridement are recommended by the guideline. However, those treatments can only be performed in the hospital in Taiwan. It is highlighted that protecting skin away from moisture is of great importance for PI prevention and wound healing. In some instances, the addition of a traditional pressure relief mattress will be suggested, such as a gel pad or APAM. In the case study, routine wound care and an APAM initially improved wound healing. After 5.5 months, however, no further wound improvement was found, an outcome similar to those reported in the literature. Thus, the addition of a traditional mattress for PI may only slightly assist with wound healing. On the other hand, the mechanism of an air-fluidized bed uses flowing sand in the mattress that allows the human body to “float” in such a way that PIs can be prevented and wound healing [7] can be promote [8]. For bedridden residents, such beds have been identified as the most useful devices for PI prevention. However, they have the disadvantages of weight and expense. This case study involved a bedridden resident with hemiplegia who was suffering from a Stage 3 PI. Previous research has suggested that a Stage 2 PI wound takes an average of 1 month to heal and that a Stage 3 or 4 PI wound takes up to 4 months. The wound size of the resident decreased after receiving routine wound care and the use of a traditional air mattress for 5.5 months, but the wound depth did not change significantly. The process of wound healing has 4 phases: hemostasis, inflammation, proliferation, and maturation, and the typical period of wound healing is 4 or 5 weeks. The phase of hemostasis or inflammation takes less time, but the phases of proliferation and maturation persist the longest to create a skin scab. The scab may need up to 9 months to fall off from the healed skin. If a wound does not heal for more than 30 days, it is referred to as a chronic wound; most PI wounds at Stage 2 or above are considered chronic wounds [19]. Factors associated with prolonged wound healing include desiccation, infection, abnormal microbial increase, necrosis, pressure, and trauma. Theoretically, pressure on a part of the human body can inhibit or block blood flow that would provide oxygen or nutrients to the wound, thus prolonging the wound healing time [20]. With 5.5 months of wound care in the nursing home, the wound size of the resident in this study was reduced to 2 × 2.5 × 3 cm^3^ and remained at Stage 3. Since no further improvement was seen, the decision was made to replace the traditional APAM for the AI mattress; the routine care was kept. The AI mattress is programed to complete a reading with modifications to the cells every 60 min or every time the resident switches position. The pressure would be redistributed after identifying the bony prominences at risk. Research showed that with appropriate use of wound care and nutritional support, removing pressure from a wound is critical and effective for wound healing [21]. Removing pressure from the PI wound can increase blood circulation around the wound, accelerate the elimination of wound waste, increase wound immunity, and provide a better healing environment. Changes in the microclimate of the wound may also be a contributory factor [9]. The environment of skin under pressure may feature high humidity and heat. Once the pressure has decreased, the microclimate of the wound area may become more amenable to wound healing Microclimate comprises of temperature, humidity, and airflow [9]. When the resident encountered the use of medical or nursing care procedures, such as dressing, diaper changes, repositioning, wound care and more, his microclimate changes. Microclimate can affect the degree of soft tissue deformation and responses. In turn, the skin condition may change [6, 9].

In addition to wound healing, the staff reported that the sleep quality of the resident improved significantly after using the device, the frequency of moans for unknown reasons decreased, and sleep time was prolonged. The increased comfort could be attributed to improvement in the PI wound. Several instances of eczema on the back of the resident was found, which may be related to moisture-associated skin damage (MASD) [22]. The changing of clothing can lead to friction and shear which can damage the skin. The moisture from sweating may change the microclimate of the skin [18]. As the pressure is relieved by the AI mattress the microclimate may change, and eczema may improve.

In the examination of pressure on the scapula, sacrum, and hips (Table 1) it showed significantly higher pressure on the affected(hemiplegic) side than on the unaffected side of the body. This finding is similar to Chung’s study in 2006. The study was to compare the differences of skin pressure between hemiplegia and healthy persons in different position [23]. It is possible that muscle in the hemiplegic side is too weak to support body weight. Therefore, the hemiplegic side is at greater risk to develop PI than the unaffected side. Furthermore, even the nursing assistants tended to decrease the time spent by the resident lying on the hemiplegic side (left side), which would explain why this was the lowest average time. However, the finding still indicates that the pressure of the hemiplegic side is still higher than the unaffected side.

On the other hand, the nursing assistants may have tended to settle the resident in the supine position or sitting up in a 30° position [23]. It is possible that this is the most comfortable and natural posture. The other possibility is that diaper changes, nasogastric tube feeding, and wound changes require lying in the supine position or sitting on the bed at 30°. Because the staff were aware that the AI device was being used, they probably believed that it was removing most of the pressure. This leads to the increased length of time spent by the resident in supine or sitting up in a 30° position in weeks 3 and 4.

There was no deterioration in wound healing or the development of new PI sites. Moreover, the total number of turns to different orientations tended to be similar, which is beneficial for consistent care in nursing homes, suggesting a positive effect of the AI mattress on nursing quality and management.

Unfortunately, the resident was transferred to the hospital because of renal problems. Consequently, it was not possible to determine whether the PI wound had fully healed. However, the depth of the PI wound persisted throughout. Friction and shear are typically identified as the major factors leading to the development of PIs by the NPUAP [2], and both are regarded as major risk factors by the Braden scale. Friction and shear can be generated by changing diapers, uncovering gauze, and incorrect turning so that the healed wound tears repeatedly. Because these improper care behaviors occur easily, freshly healed tissues can crack, resulting in prolonged proliferation and remodeling delay. Furthermore, residents with multiple illnesses, as in this case, have difficulty following care plans because of frequent visits between hospitals and nursing homes.

## Conclusion

This is a case study, though the use of an innovative AI seemed to be able to improve PI healing, a single case can just be a reference for other similar cases. The assumption was that if compressed skin could be not only redistribution(relieved) based on skin pressure automatically, but the pressure can be relieved in the smaller portion than the traditional air mattress. Then, PI could be prevented, and wound healing could be accelerated. Even though the total frequency of repositioning and wound care routine and position change frequency were not changed, the resident’s PI wound showed greater improvement with the use of the AI mattress than with traditional routine care. However, a larger sample size is needed to validate the effectiveness of the device and will be necessary to determine whether the device can be used to reduce the frequency of position changes needed. In the future, comparisons with other types of mattresses such as mattresses designed for changes of pressure to the body, or the mattress with constant low pressure over the whole body should be done. These studies should be done to allow for the correct allocation of mattresses for the residents with PI.

### Electronic supplementary material

Below is the link to the electronic supplementary material.

Supplementary Material 1

## Data Availability

The manuscript, including all relevant raw data, will be freely available to any scientist wishing to use them for non-commercial purposes, without breaching participant confidentiality. The data can be obtained through contacting the correspondent author.
